# Impact of cooking on the antioxidant activity of spice turmeric

**DOI:** 10.29219/fnr.v63.3451

**Published:** 2019-05-31

**Authors:** Jian-Long Sun, Hong-Fang Ji, Liang Shen

**Affiliations:** 1 Institute of Biomedical Research, Shandong University of Technology, Zibo, Shandong, People’s Republic of China; 2 Zibo Key Laboratory for Neurodegenerative Diseases Drug Development, Shandong Provincial Research Center for Bioinformatic Engineering and Technique, School of Life Sciences, Shandong University of Technology, Zibo, Shandong, People’s Republic of China

**Keywords:** curcuminoids, boil, roast, antioxidant activity, oxidative stress, PC12 cells

## Abstract

Curcuminoids, as the main ingredient of turmeric, are popularly used in food additives and condiments, and are widely accepted to be beneficial for human health for their antioxidant activity. However, curcuminoids are highly susceptible in terms of thermal-induced degradation, and curry is usually boiled, roasted, or fried in the use of food additives and condiments. Thus, it is interesting to explore the effect of cooking on the antioxidant activity of curcuminoids. In the present study, the total antioxidant capacity (T-AOC) of cooked curcuminoids (boiled curcuminoids, roasted curcuminoids, and fried curcuminoids) processed through three heating conditions, and their protective effects against oxidative damage to rat pheochromocytoma (PC12) cells, a well-established neuronal model, were evaluated. It was found that cooking slightly lowered the T-AOC of curcuminoids, with boiled curcuminoids being relatively stronger than roasted curcuminoids, and fried curcuminoids being the weakest form. Both boiled and roasted curcuminoids could significantly improve cell viability, mitigate intracellular accumulation of reactive oxygen species and reduce malondialdehyde activity, reduce caspase-3 and caspase-9 protein expression, and increase superoxide dismutase activity of PC12 cells compared with the control group. In comparison with parent curcuminoids, the protective effects of cooked curcuminoids got relatively lower overall, with boiled curcuminoids being relatively stronger than roasted curcuminoids. In conclusion, the cooked curcuminoids, including boiled and roasted forms, still have antioxidant and neuroprotective activity.

## Popular scientific summary

- Curcuminoids are the main ingredients of spice turmeric, which is usually heated during cooking.
- The present study indicated that the cooked curcuminoids still possessed antioxidant activity.

As the main ingredient of spice turmeric, curcuminoids are popularly used as food additives. Curcuminoids are responsible for the bright-yellow color of turmeric and thus are also used as a food coloring. It is widely recognized that curcuminoid supplementation is beneficial to human health and prevents many types of diseases in view of their multiple biological activities, including antioxidant, antiinflammatory, antibacterial, antiviral, antidiabetic, anticancer effects, etc. (1–4).

As we know, when curcuminoids are used as food additives or spice, they are usually processed by boiling or frying in oil during cooking. However, curcuminoids have the characteristic of low stability and degrade readily (5–8). Thus, it is interesting to evaluate the biological activities of boiled and roasted curcuminoids by comparing with the parent form.

Curcuminoids are natural antioxidants and can attenuate oxidative stress through scavenging reactive oxygen species (ROS) (9–11). The antioxidant activity of curcumin is a primary mechanism that explains the majority of their beneficial effects. In recent years, a series of *in vitro* and *in vivo* animal models, as well as epidemiological studies, have supported the benefits of curcumin in preventing and combating Alzheimer’s disease (AD) (12–19). Oxidative stress-induced cell injury via apoptosis or necrosis is regarded as the principal pathogenic factor of AD and other neurodegenerative diseases (20–22). Therefore, in this study, three conditions were employed to mimic boiling and roasting of curcuminoids during cooking. We studied the total antioxidant capacity (T-AOC) of boiled curcuminoids, roasted curcuminoids and fried curcuminoids, and the protective effects on the oxidative damage to PC12 cells.

## Materials and methods

### Materials

The rat pheochromocytoma PC12 cell line was purchased from the Type Culture Collection of the Chinese Academy of Sciences (Shanghai, China). The reagents for cell culture, including penicillin–streptomycin, Nutrient Mixture F-12 Ham were purchased from Sigma (St. Louis, MO, USA). Horse serum (HS) and fetal bovine serum (FBS) were from Gibco (import). H_2_O_2_ was purchased from Shuangshuang Chemical Co., Ltd. (Yantai, China). Curcuminoids (mainly containing curcumin, Curcuminoids contain a mixture of curcumin; demethoxycurcumin and bidemethoxycurcumin. If available mention their proportion; How does it differ from pure curcumin that contains 98% curcuminoids? demethoxycurcumin, and bidemethoxycurcumin) were purchased from Shanghai Macklin biochemical science and Technology Co., Ltd. 1-(4,5-dimethylthiazol-2-yl)-3,5-diphenylformazan (MTT), poly-D-lysine hydrobromide, and 2′,7′-dichlorofluorescindiacetate (DCFH-DA) were purchased from Sigma-Aldrich Shanghai Trading Co., Ltd. (Shanghai, China). The T-AOC assay kit was purchased from Beyotime Biotechnology (Shanghai, China). The assay kits for malondialdehyde (MDA), superoxide dismutase (SOD), caspase-3, and caspase-9 were purchased from Nanjing Bibo Biological Technology (Nanjing, China).

### Preparation of boiled curcuminoids, roasted curcuminoids, and fried curcuminoids

The preparation of boiled curcuminoids, roasted curcuminoids, and fried curcuminoids to mimic cooking was carried out as follows. PBS (0.01 M, pH 7.4) was used to dissolve curcuminoids to 1 mg/mL in sterilized centrifuge tubes, and then heated for 1 h in water at 100°C. Freeze drier was used to obtain drug powder, and then was dissolved in dimethyl sulfoxide (DMSO), and finally filtered by millipore filters for further experiment. As for roasted curcuminoids, curcuminoids were roasted at 200°C for 1 h with an electric heating pan dryer, and then the drug powder was dissolved in DMSO, and filtered by 0.22 μm millipore filters for further study. Edible blending oil was used to dissolve curcuminoids to 1 mg/mL in sterilized glass tubes and then heated at 150°C for 10 min with collector type constant temperature heating magnetic agitator, to prepare the fried curcuminoids sample.

**Table 1:** Total antioxidant capacity of curcuminoids, boiled curcuminoids, roasted curcuminoids, and fried curcuminoids

Compounds (1 mg/mL)	Total antioxidant capacity (T-AOC)
Curcuminoids	3.71 ± 0.18
Boiled curcuminoids	2.33 ± 0.04**
Roasted curcuminoids	1.06 ± 0.16**
Fried curcuminoids	0.212 ± 0.06**

Data were shown as mean ± SD (*n* = 3). (*P < 0.05 and **P < 0.01 vs. curcuminoids).

### Total antioxidant capacity assay

The T-AOC of curcuminoids, boiled curcuminoids, roasted curcuminoids, and fried curcuminoids, was estimated with ferric reducing ability of plasma (*FRAP*) method. After preparation of the FRAP reagent, it was incubated at 37°C and was used within 2 h. A total of 5 μl of standard solution at a range of concentrations or tested sample was added to the FRAP reagent for 5 min at 37°C. Finally, the absorbance value was measured by the Multiwell microplate reader (MultiskanGo, Thermo Fisher Scientific, Vantaa, Finland) to calculate the T-AOC of the samples.

### Cell viability assay

PC12 cells were cultivated in Nutrient Mixture F-12 Ham media supplemented with 10% HS, 5% FBS, 1% (v/v) penicillin and streptomycin at a temperature of 37°C with 5% CO_2_. Cytoprotective activity of curcuminoids, boiled curcuminoids, and roasted curcuminoids on cell injury caused by H_2_O_2_ was assessed by MTT assay (18).

### ROS assay

The intracellular ROS level was estimated by means of a DCFH-DA assay. PC12 cells were pretreated with curcuminoids, boiled curcuminoids, and roasted curcuminoids for 0.5 h and then H_2_O_2_ was added for additional 6 h. Then, the PC12 cells were incubated with serum-free medium containing 10 μM DCFH-DA for 0.5 h. The relative fluorescence intensity of cells was estimated with a fluorescent microplate reader (Varioskan Flash, Thermo Fisher Scientific Oy, Vantaa, Finland), and then pictures were taken under a fluorescence microscope (Olympus IX73, Tokyo, Japan).

### MDA, SOD, caspase-3, and caspase-9 assays

Cells were incubated in a 6-well plate for 24 h. Treatment with different concentrations of curcuminoids, boiled curcuminoids, and roasted curcuminoids on PC12 cells was same as the above assay. After 12 h, cells were washed with PBS and homogenized. The homogenate was centrifuged to collect the supernatant for further study. The MDA level and SOD, caspase-3 and caspase-9 activities, were determined by means of assay kits according to the manufacturer’s instruction.

### Statistical analysis

All values obtained from three independent experiments were analyzed with SPSS (16.0) software and shown as the mean ± standard derivation (SD). The difference in different groups was compared and analyzed by one-way analysis of variance (ANOVA). The minimum significance level was defined as ^##^ *P* < 0.01, ^#^ *P* < 0.05 versus control, and **P < 0.01, *P < 0.05 versus H_2_O_2_-treated cells.

## Results

### Total antioxidant capacity

The T-AOC of curcuminoids, boiled curcuminoids, roasted curcuminoids, and fried curcuminoids was measured using the FRAP method, and was characterized by mmol Fe^2+^ per milligram of dry weight. It was found that parent curcuminoids exhibited relatively higher T-AOC than the cooked forms. The T-AOC of boiled curcuminoids was found to be higher than roasted curcuminoids, with fried curcuminoids being the weakest form.

### Protective effects of boiled and roasted curcuminoids

**Fig. 1:**
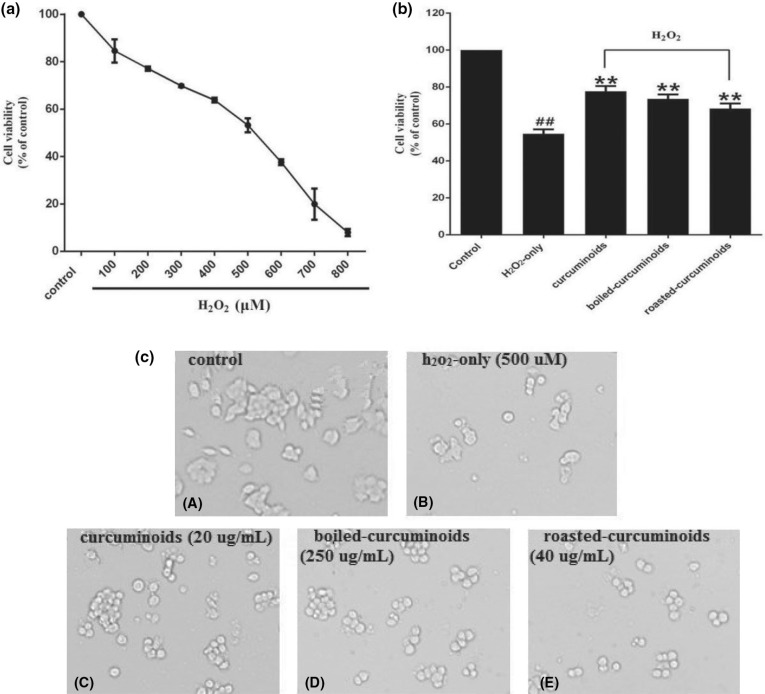
Protective effects of curcuminoids, boiled curcuminoids, and roasted curcuminoids on PC12 cells against H_2_O_2_-induced damage. Cell viabilities treated with different concentrations of H_2_O_2_ (a). The protective effects of curcuminoids, boiled curcuminoids, and roasted curcuminoids on H_2_O_2_-induced cytotoxicity to PC12 cells (b). Morphological alteration of PC12 cells (c): (A) control cells; (B) cells treated with H_2_O_2_ only; cells pretreated with curcuminoids (20 μg/mL) (C), boiled curcuminoids (250 μg/mL) (D), roasted curcuminoids (40 μg/mL) (E), and co-treated with H_2_O_2._ Data were shown as mean ± SD (*n* = 3). (^##^*P* < 0.01 and ^#^*P* < 0.05 vs. control; *P < 0.05 and **P < 0.01 vs. H_2_O_2_-treated cells).

After testing different concentrations of H_2_O_2_ to damage the PC12 cells, we found that the survival rate of PC12 cells was 53.19 ± 2.96% at 500 μM concentration of H_2_O_2_. Therefore, the 500 μM concentration of H_2_O_2_ was used in further experiments as an oxidative injury model (Fig. 1a). According to the protection experiment, we found that curcuminoids (20 μg/mL), boiled curcuminoids (250 μg/mL), and roasted curcuminoids (40 μg/mL) have a stronger protective effect than the H_2_O_2_-only group, and increased the cell viability by 23.07 ± 4.42%, 18.91 ± 1.62%, and 13.72 ± 0.87%, respectively (Fig. 1b). Therefore, the three concentrations were used for all further experiments. Morphological cell image analysis indicated that PC12 cells exposed to 500 μM H_2_O_2_ for 24 h resulted in morphological alteration and cell number reduction, while pretreating with curcuminoids or the boiled and roasted curcuminoids attenuated the damages (Fig. 1c).

### Intracellular ROS

**Fig. 2:**
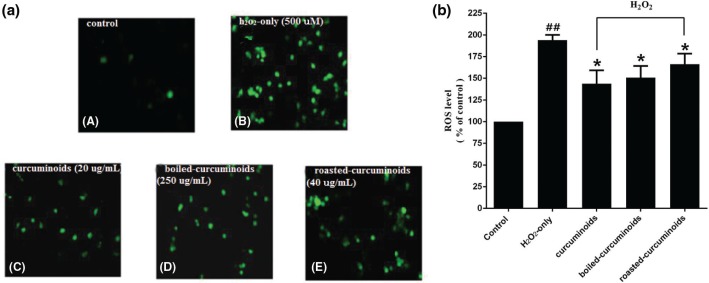
Effects of curcuminoids, boiled curcuminoids, and roasted curcuminoids on intracellular ROS level. (a) Representative images of DCFH-DA staining in PC12 cells by the inverted fluorescence microscope. The labeling letters in this experiment were identical with Fig. 1C; (b) Intracellular ROS level. Data were shown as mean ± SD (*n* = 3). (^##^*P* < 0.01 and ^#^*P* < 0.05 vs. control; *P < 0.05 and **P < 0.01 vs. H_2_O_2_-treated cells).

The intracellular ROS levels were estimated with the fluorescence probe DCFH-DA. In comparison with the control group, the intracellular ROS level in the oxidative injury group increased significantly to 194 ± 10.44% (Fig. 2a). PC12 cells pretreated with curcuminoids, boiled and roasted forms, significantly reduced ROS levels. Curcuminoids exhibited the most significant effect (143.67 ± 26.95%), followed by boiled and roasted curcuminoids (Fig. 2b).

### MDA and SOD

**Fig. 3:**
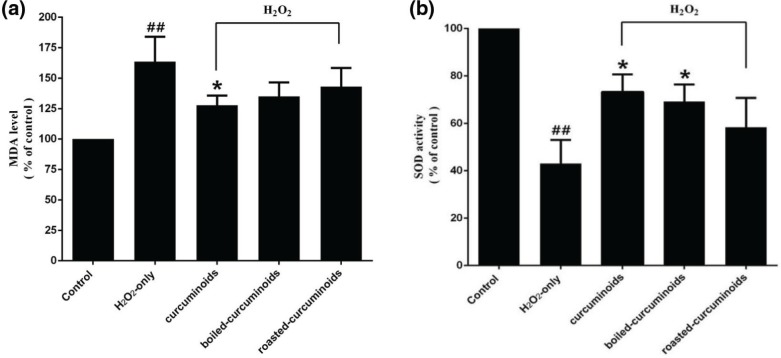
Effects of curcuminoids, boiled curcuminoids, and roasted curcuminoids on the MDA level (a) and SOD activity (b) in PC12 cells caused by H_2_O_2._ Data were shown as mean ± SD (*n* = 3). (^##^*P* < 0.01 and ^#^*P* < 0.05 vs. control; *P < 0.05 and **P < 0.01 vs. H_2_O_2_-treated cells).

PC12 cells exposed to H_2_O_2_ caused an increase in the intracellular MDA level and a decrease in the SOD activity. Pretreating cells with curcuminoids, boiled curcuminoids, or roasted curcuminoids for 0.5 h before being exposed to H_2_O_2_, clearly attenuated intracellular MDA level increase (Fig. 3a) and enhanced the SOD activity (Fig. 3b). The effect of boiled curcuminoids was relatively stronger than that of roasted curcuminoids.

### Caspase-3 and caspase-9

**Fig. 4:**
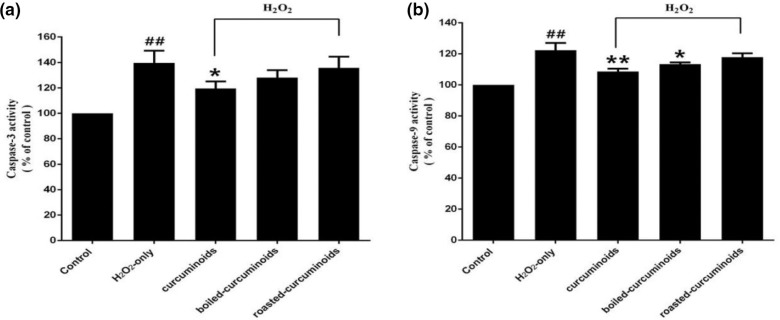
Effect of curcuminoids, boiled curcuminoids, and roasted curcuminoids on caspase-3 (a) and caspase-9 (b) activities induced by H_2_O_2_. Data were shown as mean ± SD (*n* = 3). (^##^*P* < 0.01 and ^#^*P* < 0.05 vs. control; *P < 0.05 and **P < 0.01 vs. H_2_O_2_-treated cells).

Exposing PC12 cells to H_2_O_2_ resulted in an enhancement of caspase-3 and caspase-9 activities. Pretreatment with curcuminoids, boiled curcuminoids, and roasted curcuminoids effectively suppressed an increase in caspase-3 (Fig. 4a) and caspase-9 (Fig. 4b) activities caused by H_2_O_2._

## Discussion

Food processing can regulate biological activity of plant bioactive substances (23, 24). Turmeric, with curcuminoids as the main bioactive components, is a popular food additive and condiment. However, curcuminoids readily degrade when heated, and cooking like boiling and roasting will result in the degradation of curcuminoids to a great extent (5–7, 16). The degradation products of curcumin after cooking also possess biological properties similar to the parent compound (25, 26). Curcuminoids were reported to have neuroprotective effects via reducing oxidative stress (27). Herein, we studied the T-AOC, the protective effects of boiled and roasted curcuminoids on oxidative damage to PC12 cells. Boiled curcuminoids, roasted curcuminoids, and fried curcuminoids still possess T-AOC, and the capacity of fried curcuminoids was the weakest among the three forms. Both boiled and roasted curcuminoids could reduce the content of ROS, decrease the MDA level, and increase SOD activity. Previous studies indicated that the degradation products produced after heating, which retain the main functional groups of curcumin, contribute to the various pharmacological effects of curcuminoids (5–7, 16). The boiled and roasted curcuminoids also exhibited neuroprotective ability. This may provide potential clues to understand the reduced incidence of AD after consuming curcuminoids as food additives in daily life (17). In addition, it was also found that boiled curcuminoids possessed relatively stronger antioxidant activity than the roasted and the fried form, which may arise from two factors: 1) heating increased the solubility of boiled curcuminoids in PBS and 2) higher temperatures and oxygen exposure may destroy the antioxidant functional structures of roasted and fried curcuminoids to a certain extent.

## Conclusions

In summary, the present study indicated that after processing with heating conditions mimicking cooking, boiled and roasted curcuminoids still possessed antioxidant capacities and could inhibit PC12 cellular injury induced by H_2_O_2_ through ameliorating oxidative stress, and the effect of boiled curcuminoids was relatively higher than the roasted form. More studies are encouraged to elucidate the molecular basis underlying the differences in neuroprotective activities between boiled and roasted curcuminoids.
